# Gua sha therapy for chronic low back pain: A protocol for systematic review

**DOI:** 10.1097/MD.0000000000020606

**Published:** 2020-10-02

**Authors:** Yu-wei Wang, Zhen-wen Xi, Bin Pu, Guang-yan Chen, Yu-feng Ma, Ding-long Liu, Xiao Xu

**Affiliations:** a Department of Traditional Medical Diagnosis and Treatment Center, Gansu Provincial Hospital, Lanzhou City, Gansu Province; b School of Nursing, Changzhou University, Changzhou City, Jiangsu Province, China.

**Keywords:** gua sha, non-specific chronic low back pain, protocol, systematic review

## Abstract

**Background:**

Non-specific chronic low back pain (LBP) is a debilitating disease that profoundly impacts patients’ daily physical function and quality of life. Gua sha therapy, as an easy-to-use and noninvasive complementary modality, has been widely used clinically in patients with non-specific chronic LBP. The aim of this study is to test the potential benefits and harms of gua sha therapy on patients with non-specific chronic LBP.

**Methods:**

Ten English databases, 3 Korean databases, 6 Chinese databases, 1 Japanese database, and 2 Brazilian databases will be searched from their inception to September 2019. Randomized controlled trials will be included if gua sha therapy was used as the sole treatment or as a part of combination therapy with other treatments in patients with non-specific chronic LBP. Two reviewers will independently extract the data and assess the methodological quality using the Cochrane criteria for risk of bias. The meta-analysis will be performed using Review Manager 5.3 software.

**Results:**

The findings of this systematic review will be published in a peer-reviewed journal.

**Conclusion:**

This systematic review will provide more evidence regarding the clinical usage of gua sha therapy for non-specific chronic LBP.

**Trial registration number:**

CRD42019134567

## 1 Introduction

Non-specific low back pain (LBP) is commonly defined as pain or discomfort localized in one or both lower limbs without definitive reason (such as ankylosing spondylitis, osteoporosis, inflammatory disorders, spinal fracture or surgery, infection, and spinal deformity), which persists for at least 1 day.^[1]^ Non-specific LBP that lasts >12 weeks is defined as non-specific chronic LBP.^[2]^ Recent epidemiological surveys show that approximately 70% of US adults experience LBP, and half have an episode of non-specific chronic LBP by the third decade of life.^[3]^ In Germany, data shows that approximately 25% young adults experience non-specific LBP by age 30, with a 1-year recurrence rate as high as 70%.^[4]^ Non-specific chronic LBP, as a major public health issue in both developed and developing countries, is a leading cause of sickness absenteeism from the workplace and a heavy economic burden for individuals, employers, and health insurers.^[5]^ The American Institute of Medicine has estimated that the annual direct healthcare costs related to non-specific chronic LBP treatment have been up to $34 billion.^[6]^ The Medical Utilisation and Consumption Study has also reported that non-specific chronic LBP is one of the leading reasons for seeking outpatient medical care in the Republic of Korea.^[7]^ Additionally, non-specific chronic LBP adversely influences patients’ social functioning, decreases work performance and income, and increases individual and family stress.^[8]^

The 2018 international evidence-based recommendation suggested nonopioid analgesics, such as non-steroidal anti-inflammatory drugs (NSAIDs), and opioid medications as pharmacologic therapy options for the management of non-specific LBP.^[9]^ NSAIDs might yield beneficial effects in relieving pain, suppressing inflammation, and improving daily physical function for non-specific LBP patients. However, various side effects are often associated with the long-term oral use of NSAIDs, such as peptic ulcers, increased cardiovascular risk, and renal malfunction.^[10]^ Opioids are frequently prescribed analgesic drugs for relieving non-specific LBP at emergency departments in the United States.^[11]^ Unfortunately, only very low-quality evidence suggests a better analgesic effect of opioid medication than placebos for non-specific LBP.^[12]^ Moreover, short- and long-term use of opioid medications is often associated with constipation, dizziness, psychological distress, falls, and fractures.[^13^,^14^] Given these limitations of pharmacological therapies, it is unsurprising that 1 study showed 77.3% of non-specific LBP patients in Switzerland chose to consult a complementary and alternative medicine (CAM) at a local pain center.^[15]^ Furthermore, the American College of Physicians has revised its recommendation guidelines to suggest CAM treatment options for the management of non-specific LBP.^[16]^

Manipulative and manual therapy (MMT), a costly and time-consuming CAM technique, is popular among Asian patients. Different styles of MMT exist for the management of musculoskeletal system diseases,[^17^,^18^,^19^] of which gua sha is one of the most common.^[20]^ Gua sha therapy is also known as cao gio, khoud lam, and ga sal in different Asian countries.^[21]^ In ancient Chinese textbooks, “sha” refers to the red, millet-sized rash associated with blood stasis, while “gua” means instrument-assisted scraping at specific acupuncture points and meridians.^[22]^ Thus, gua sha therapy uses a variety of smooth-edged instruments (such as Chinese soup spoons, buffalo horn, and coins) to bring therapeutic petechiae to the body's acupuncture points and surface meridians by press-stroking.^[21]^ Gua sha therapy is a widely used, ancient, noninvasive healing technique in East Asia and communities of Asian immigrants. Recently, a cross-sectional study in Taipei City revealed that gua sha therapy was the third most widely accepted MMT for the management of musculoskeletal pain, besides acupuncture–moxibustion therapy and Chinese tui na therapy.^[23]^ Moreover, another cross-sectional study found that approximately 25% of the general population in a Hong Kong community received gua sha therapy from CAM practitioners as a main modality to manage pain-related conditions.^[24]^

According to traditional Chinese medicine theory, LBP belongs to the bi syndrome. Kidney qi insufficiency and blood stasis are key to pathogenesis of LBP. Gua sha therapy may effectively remove du meridian obstructions, replenish yang, remove the blood stasis, and promote blood circulation. Moreover, several studies have contributed to explaining the possible biological mechanism of gua sha therapy for non-specific LBP. According to Jiang et al,^[25]^ gua sha therapy can prohibit local immuno-inflammatory responses via reducing serum levels of interleukin-1 in a rat model of LBP. Yang et al^[26]^ found that a 3-week gua sha therapy course could reduce the levels of substance P, neuropeptide Y, inflammatory cytokine phospholipase A2, and prostaglandin E2 in the dorsal root ganglion in a rat model. Furthermore, Ding and Chen^[27]^ identified 15 potential biomarkers and 6 metabolic pathways as gua sha-targeted molecules in a rat model of LBP, using urine metabolomics analysis.

Several systematic reviews have tested the safety and effectiveness of gua sha therapy on insomnia,^[28]^ musculoskeletal pain,^[29]^ and perimenopausal syndrome.^[30]^ Moreover, gua sha therapy, as a rapid, easy-to-use, and noninvasive complementary modality, has been widely used clinically in patients with non-specific chronic LBP. Nevertheless, to the best of our knowledge, no systematic review has specifically focused on gua sha therapy for non-specific chronic LBP. Therefore, this study aims to test the potential benefits and harms of gua sha therapy on patients with non-specific chronic LBP.

## 2 Methods

### 2.1 Protocol and registration

This protocol will be structured in accordance with the standardized 27-item checklist PRISMA-P framework. In addition, this protocol is also registered with PROSPERO (registration # CRD42019134567).

### 2.2 Study type and eligibility criteria

Only randomized controlled trials regarding the effectiveness and safety of gua sha therapy for non-specific chronic LBP will be included in this research. Other types of research (such as observational studies, pre-clinical cell and animal studies, case reports, bibliography research, and quasi-clinical trials) will be excluded. The language is not limited. The eligibility criteria will strictly comply with PICOS (participants–interventions–comparisons–outcomes) principles. The details of eligibility criteria following PICOS principles are shown in Table 1.

**Table 1:**
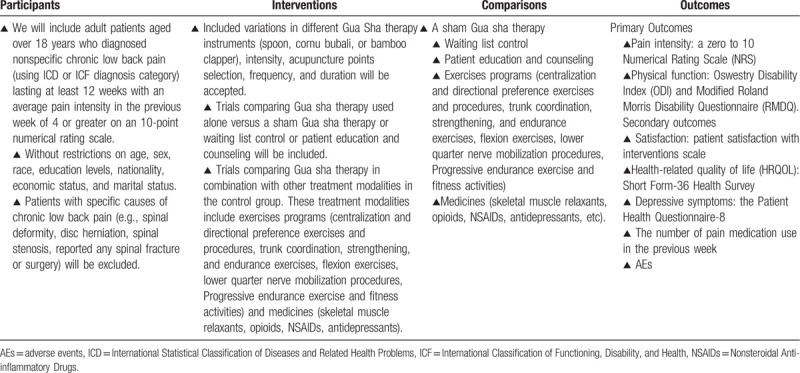
Participants, interventions, comparisons, and outcomes of this systematic review.

### 2.3 Search methods

#### 2.3.1 Electronic searches

We will search the following databases from their inception until September 2019, without language restrictions.

(1) English databases: PubMed, the Cochrane Central Register, AMED, EMBASE, the Cochrane Complementary and Alternative Medicine Group Trials Register, the Cochrane Pain and Anaesthesia Group Trials Register, the Cochrane Orthopaedics and Trauma Group Trials Register, the Cochrane Rheumatology Group Trials Register, Scopus and Web of Science.
(2) Korean databases: OASIS, Korean Medical Database and Korea Citation Index.
(3) Chinese databases: Chongqin Weipu Vip, CHKD, CNKI, Sino-Med and WanFang data.
(4) Japanese database: CiNii.
(5) Brazilian databases: Descritores em Ciências da Saúde and Coordenação de Aperfeiçoamento de Pessoal de Nível Superior.

Initially, bioinformationists in our research team will formulate retrieval strategies. Then, an external experienced librarian will scrutinize these search strategies carefully with a standard strategies checklist. The formal search terms will include: coining, skin-scraping, pressure-stroking, cao gio, kerok, kerokan, kos khyal, ga-sal, guasha, khoud, low back pain, back pain, backache, and lumbago. Some search terms will be revised slightly for each electronic database. Table 2 shows the search strategy for PubMed.

**Table 2:**
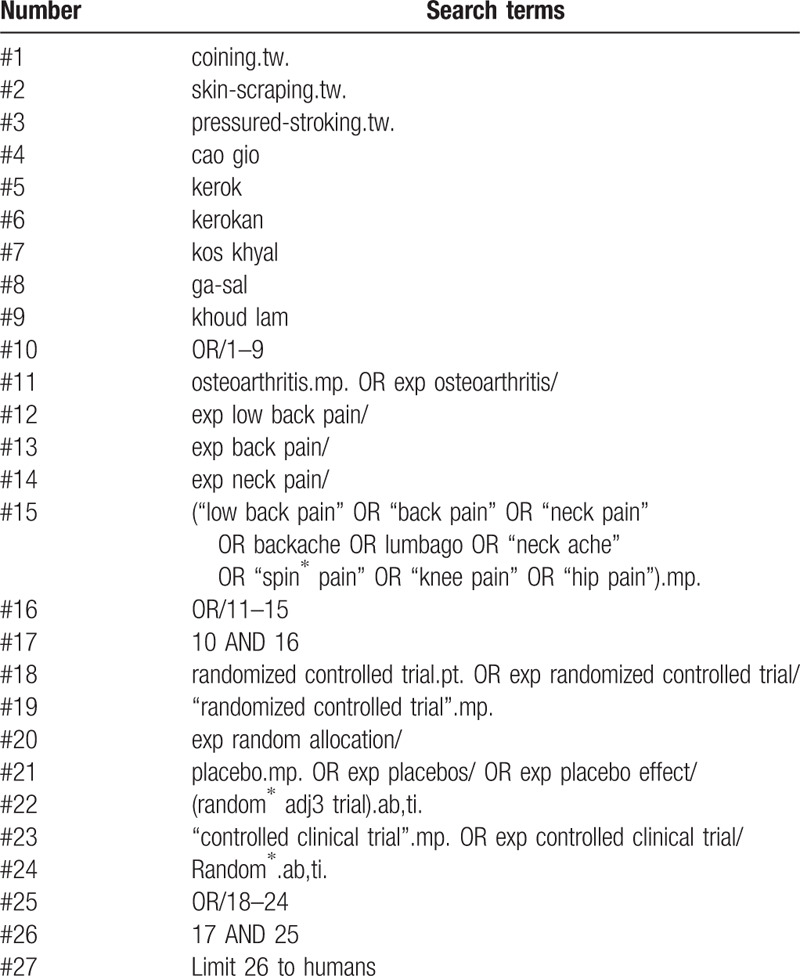
PubMed search strategy.

#### 2.3.2 Searching other resources

Firstly, some important clinical trial registry platforms (such as ClinicalTrials.gov, EU-CTR, ISRCTN, IRCT, UMIN-CTR, and ReBec) will be electronically searched. Secondly, specialist resources including the Research Council for Complementary Medicine (https://www.rccm.org.uk/) and American Holistic Nursing Association (https://www.ahna.org/) will be contacted to identify grey literature. Thirdly, some important gua sha device companies (Qinghe Chunol Medical Device Co., Ltd.; Shinylink Industrial Inc.; and Leosense Technology Company) will also be consulted for recent unpublished or updated data. Fourthly, we will manually search conference proceedings published in some important Chinese journals.

### 2.4 Data collection and analysis

#### 2.4.1 Selection of studies

Initially, 2 independent reviewers (Y-WW and Z-wX) who are experts in CAM will electronically or manually search relevant databases and journals for suitable studies. Then, all retrieved articles will be exported to EndNote X8.2 library (Thomson Reuters), a reference management software package. To prevent omissions, the results of the search process will be cross-checked repeatedly by two reviewers. After deleting duplicated records with the help of EndNote's auto-duplication function, two independent reviewers (Y-wW and Z-wX) will scrutinize each study (titles, keywords, and abstracts) and rate included trials as “relevant,” “marginally relevant,” or “unclear” based on the pre-established evaluation criteria. The full text of studies ranked as marginally relevant will be read carefully to make a decision whether to retain them. At this stage, the reasons for excluding each will be noted. If disagreement occurs at any stage of the selection process, 2 independent reviewers will resolve it through discussion. A calculated Cohen Kappa value will be used to assess the inter-rater reliability between the 2 reviewers in study selection. If necessary, the third reviewer (D-LL) will be invited as an arbitrator to make a final decision on discrepancies that cannot be solved after the negotiation between 2 reviewers.

#### 2.4.2 Data collection and management

Two data extractors (Y-wW and Z-wX) will independently extract data from each quantitative study in 2 separate spreadsheets, as recognized and recommended by the University of York CRD guidelines. A pilot data extraction will be conducted on a sample of 5 included eligible trials by 2 reviewers independently. In order to ensure consistency and agreement between data extractors, Cohen Kappa value will be calculated. The data to be extracted will include the following.

(1) Basic information: Author names and countries, publication year, title of included trial, publication journal, and funding support.
(2) Basic characteristics: Age, sex, sample size, number of participants in each group and diagnosis.
(3) Details of treatment in the intervention group: Materials used for guasha, types of guasha, duration of guasha therapy, treatment acupuncture points, reasoning for acupuncture point selection, CAM practitioner qualifications.
(4) Details of treatment in the control group.
(5) Outcomes.
(6) Methodological assessment.

Any disagreement in the data extraction procedure will be settled by discussion between 2 reviewers (Y-wW and Z-wX) or input from a third senior reviewer (D-lL). All extracted data will be entered into Review Manager (RevMan) 5.3 software from the Cochrane Collaboration.

#### 2.4.3 Addressing missing data

We will try our best to contact each trial's corresponding authors (via email or telephone) for further information if the data are insufficient or ambiguous.

#### 2.4.4 Quality appraisal

**Figure 1:**
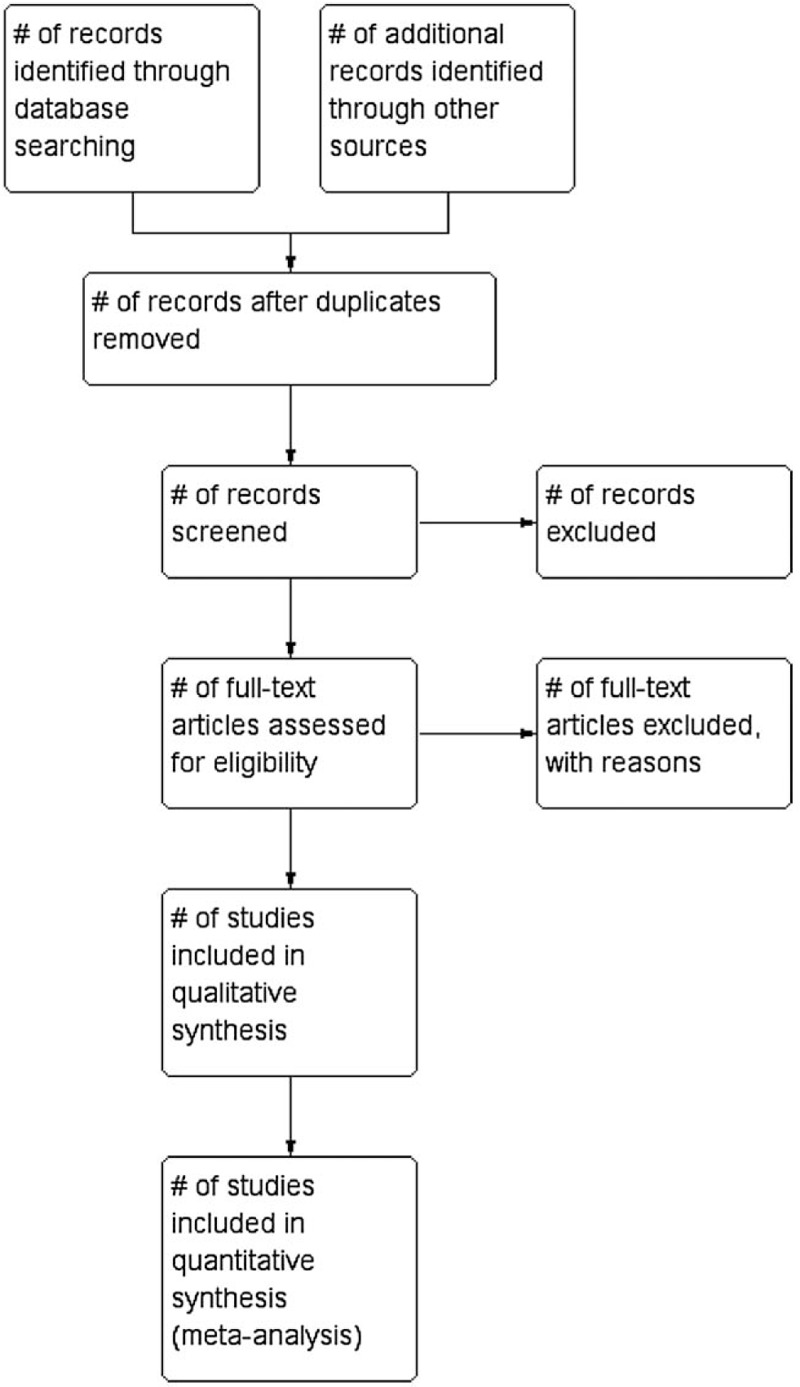
PRISMA-compliant flow chart.

The quality appraisal will be conducted by 2 independent reviewers (Y-wW and Z-wX) according to the Cochrane Collaboration's tool. Each included risk of bias domain for each included study will be summarized in a “quality assessment” Fig. 1 (with green representing low risk of bias, red representing high risk of bias, and yellow representing unclear risk of bias), using RevMan 5.3. The following domains will be assessed critically.

(1) Performance bias: It is difficult to blind the participants or experimenters during gua sha therapy. Thus, we will rate “uncertain risk” in the domain of performance bias.
(2) Attrition bias: We will assess whether drop-out rate and incomplete outcome data are reported in the clinical trial.
(3) Reporting bias: We will check previously published study protocols to identify any pertinent analyses unreported in the formal clinical trial.
(4) Selection bias: We will check whether the clinical trial adequately reports the methods of random sequence generation and allocation concealment.
(5) Detection bias: We will assess whether the clinical trial adopted outcome assessor blinding.
(6) Other bias: We will also check: baseline imbalance between intervention and control groups (age, sex, education levels, nationality, economic status, marital status, back pain history, medication usage, physical function, depression status, pain intensity, or disease severity), sample size calculation, conflicts of interest, and sources of funding support.

In most cases, 2 reviewers (Y-wW and Z-wX) will resolve any disagreements via a consensus meeting. However, if disagreements cannot be resolved after negotiation, the third reviewer (D-lL) will make the final decision regarding the methodological quality of included trials.

#### 2.4.5 Data analysis

Initially, we will assess results of the data abstraction process from 2 perspectives. Firstly, we will check whether the number of included trials meet the requirements of quantitative data analysis. Secondly, we will check whether included studies are adequately clinical homogeneous (such as in the demographic characteristics of LBP patients, variations of interventions and comparators, risk of bias, and outcome measures). If insufficient studies or significant clinical heterogeneity or variability do exist, we will perform a brief narrative form instead of meta-analysis for each outcome. Otherwise, we will adopt a quantitative meta-analysis using RevMan 5.3.^[31]^

Since different tools measure patients’ pain levels, we will express the outcome of numerical rating scales as a standardized mean difference with 95% confidence intervals. For measuring other continuous data (Oswestry Disability Index, Modified Roland Morris Disability Questionnaire, patient satisfaction with interventions scale, health-related quality of life, and depressive symptoms), the mean difference of the effect size will be applied in the meta-analysis. For measuring dichotomous data (pain medication use in the previous week and adverse events), we will calculate results using risk ratio with 95% confidence intervals.

#### 2.4.6 Assessment of heterogeneity

The *I* ^2^ statistic (test levels *α* = 0.1) will be used to measure the heterogeneity of each included study. *I* ^2^ ≤ 25% will be classified as low heterogeneity, 25% < *I* ^2^ < 75% will be classified as moderate heterogeneity and *I* ^2^ ≥ 75% will be classified as high heterogeneity.^[32]^ If *I* ^2^ < 50% and *P* > .1, we will carry out quantitative meta-analysis with the use of a fixed effect model; otherwise, if *I* ^2^ ≥ 50% and *P* < .1, the data will be synthesized using a random effects model, and additional subgroup or sensitivity analysis will be performed to determine the underlying causes of heterogeneity.

#### 2.4.7 Additional analysis

##### 2.4.7.1 Subgroup analysis

Subgroup analysis will be performed according to:

(1) Place of residence (inpatient departments vs outpatient departments vs community settings).
(2) Different control interventions (sham gua sha therapy vs waiting list control vs patient education and counselling vs exercises programs vs medicines).
(3) Different materials of gua sha instruments (spoon vs bamboo clapper), and different frequencies of gua sha therapy (≤3 times/wk vs >3 times/wk).
(4) Different durations of follow-up (<12 weeks vs 12–24 weeks vs >24 weeks).
(5) Different study designs (trials comparing gua sha therapy in combination with other treatment modalities in the control group or trials comparing gua sha therapy used alone).

##### 2.4.7.2 Sensitivity analysis

To test the robustness and reliability of the outcome measures, we will perform sensitivity analysis by omitting any study with high risk of bias or low sample size, and changing the statistical model.

#### 2.4.8 Assessment of reporting bias

Funnel plots drawn with RevMan 5.3 will be used to assess potential publication bias (at least 10 studies).^[33]^

### 2.5 Grading the quality of evidence

We will use the GRADEpro web tool (https://gradepro.org/) to assess the quality of evidence of each key outcome. The results of each key outcome will be categorized using four grades: high (⊕⊕⊕⊕), medium (⊕⊕⊕⊖), low (⊕⊕⊖⊖), or very low (⊕⊖⊖⊖).^[34]^

### 2.6 Ethic approval

The privacy of individual patients is not involved in this systematic review, so no ethical approval is required.

## 3 Discussion

Non-specific chronic LBP is a debilitating disease that profoundly impacts patients’ daily physical function and quality of life. According to the 2018 international evidence-based recommendation, non-opioid analgesics and opioid medications are the main available treatment options. While pharmacological agents may assist non-specific chronic LBP, they can have serious adverse effects, including constipation, dizziness, psychological distress, falls and fractures. Gua sha therapy, as an easy-to-use, noninvasive complementary intervention, has been proven to exert anti-inflammatory and pain-easing effects on non-specific LBP in in vivo studies. Thus, gua sha therapy could provide CAM practitioners another treatment option for the management of non-specific LBP.

This systematic review may have some limitations. Firstly, due to the nature of gua sha interventions, studies may be unable to blind clinical practitioners and participants. Secondly, considering that gua sha originated in Asian countries, doctor–patient relationships and high expectations for treatment success cannot be avoided.

## Author contributions

The study was conceptualized Yu-wei Wang. The methodology was performed by Zhen-wen Xi, Bin Pu, and Xiao Xu. The manuscript was drafted by Yu-wei Wang, Guang-yan Chen, and Yu-feng Ma. Ding-long Liu revised and edited this article. Yu-wei Wang, Zhen-wen Xi, Bin Pu, Guang-yan Chen, Yu-feng Ma, and Ding-long Liu, approved this final publication.
